# My Fear Is Not, and Never Will Be, Your Fear: On Emotions and Feelings in Animals

**DOI:** 10.1007/s42761-021-00099-x

**Published:** 2022-03-10

**Authors:** Mariska E. Kret, Jorg J. M. Massen, Frans B. M. de Waal

**Affiliations:** 1 Cognitive Psychology Unit, Institute of Psychology, Leiden University, Leiden, The Netherlands; 2 Comparative Psychology & Affective Neuroscience Lab, Cognitive Psychology Department, Leiden University, Leiden, The Netherlands; 3 Leiden Institute for Brain and Cognition (LIBC), Leiden, The Netherlands; 4 Animal Behaviour and Cognition, Department of Biology, Utrecht University, Utrecht, The Netherlands; 5 Psychology Department, Emory University, Atlanta, GA USA

**Keywords:** Emotion, Feeling, Consciousness, Anthropomorphism, Evolution

## Abstract

Do nonhuman animals (henceforth, animals) have emotions, and if so, are these similar to ours? This opinion piece aims to add to the recent debate about this question and provides a critical re-evaluation of what can be concluded about animal and human emotions. Emotions, and their cognitive interpretation, i.e., feelings, serve important survival functions. Emotions, we believe, can exist without feelings and are unconsciously influencing our behavior more than we think, and possibly more so than feelings do. Given that emotions are expressed in body and brain, they can be inferred from these measures. We view feelings primarily as private states, which may be similar across closely related species but remain mostly inaccessible to science. Still, combining data acquired through behavioral observation with data obtained from noninvasive techniques (e.g., eyetracking, thermography, hormonal samples) and from cognitive tasks (e.g., decision-making paradigms, cognitive bias, attentional bias) provides new information about the inner states of animals, and possibly about their feelings as well. Given that many other species show behavioral, neurophysiological, hormonal, and cognitive responses to valenced stimuli equivalent to human responses, it seems logical to speak of animal emotions and sometimes even of animal feelings. At the very least, the contemporary multi-method approach allows us to get closer than ever before. We conclude with recommendations on how the field should move forward.

## Various Positions on Animal Emotions and Feelings

Two decades ago, a symposium was held in Amsterdam with many luminaries of affective science. The symposium’s title, *Feelings and Emotions*, generated lively debate about the definition of both concepts. Some speakers considered them merely two sides of the same coin, whereas others saw a sharp contrast. Damasio (2004, pp. 50, 52) stressed how emotions are “bioregulatory reactions” that prepare the organism for adaptive behavior (cf. Frijda, 2010), whereas feelings are the mental representations of the physiological changes that occur during an emotion. In the current article, we adhere to these two definitions. Most contemporary researchers do not deny the existence of emotions in animals*.* Disagreements mostly concern feelings. It is important therefore, to discuss the terminology to describe research findings and how far researchers may go in interpreting their data (LeDoux, 2017; Mobbs et al., 2019). Since there is little consensus in the literature, we will start off reviewing the views of some of the key researchers. See Table 1 for a summary of the terminology used in this paper.

**Table 1:** Clarification of our terminology

Terms	How the terms are used in this article	Species-specific aspects	
		Animals	Humans
Emotion primitives	Functional and adaptive properties, such as approach or avoidance behaviors which are observable, and which are expressions of internal brain (or emotion) states	Simple organisms can show emotion primitives as these have clear survival functions	Also humans can have emotion primitives
Survival functions	A subcortical defensive survival circuit centered on the amygdala which initiates defensive behaviors in response to threats	Present in all vertebrates	
Emotions	Emotions are “bioregulatory reactions” that prepare the organism for adaptive behavior. They can exist without feelings and are unconsciously influencing behavior more than we think, and possibly more so than feelings do	Via observation of the expressions or by taking physiological measures, animal emotions can be inferred	Without being aware of it, emotions can have a large impact on humans behavior and decisions
Feelings	Feelings are the mental representations or cognitive interpretations of the physiological changes that occur during an emotion	We suggest experiential similarity between related species, but for the moment animal feelings remain inaccessible	Generally seen as what people verbally report as their subjective experience of emotion, although, for reasons outlined in the text, this may not be accurate
Evolutionary parsimony	When related species show similar behavior under similar circumstances, this is likely driven by similar psychological processes	A difficulty is that there is no hard line or golden standard that states when species are closely related enough	
Cognitive parsimony	The simplest possible cognitive process should be assigned to observed behaviors	This is applied most often when interpreting the behavior of nonhuman animals	This is seldomly applied when interpreting human behavior

Panksepp’s (2011) view on animal emotions was that all mammals share neural pathways that are linked to emotions. Therefore, advances in the study of neurobiology and neuroscience will help in understanding the biology and psychology of emotion. According to him, the awareness of both one’s own and others’ emotions^1^ is an intrinsic function of the brain, shared homologously by all mammalian species. We can study these functions in other animals via clever experimental paradigms using brain measures (Panksepp, 2005).

Anderson and Adolphs (2014) define emotions as internal brain states and view emotional behaviors as a class of behaviors that express these internal emotion states. These states exhibit general functional and adaptive properties, called “emotion primitives,” that apply across species. These properties can be studied even in evolutionarily distant organisms such as the fruit fly, allowing functional dissection of their mechanistic bases and tests of their causal relationships to behavior. The authors’ empirical approach circumvents the question of whether or not animals have feelings.

Barrett (2006) states that top-down conceptual knowledge is essential to shape emotions in the social world. She makes a distinction between hominids (great apes and humans) and the rest of the animal kingdom. Great apes have dense interconnectivity between subcortical and cortical areas and among cortical areas (but see Stacho et al., 2020, for similar findings in birds), the functional consequences of which should not be ignored. To Barrett (2012), emotions are not mechanistically present: They can be expressed in different ways and don’t have distinct neurophysiological correlates. Barret’s approach sheds light on the meaning-making process, with a research focus on humans and the way feelings are being constructed. Since psychological research relies mostly on self-report, its findings concern, in our terminology, feelings rather than emotions. Accordingly, constructional theories, such as Barrett’s, mostly concern feelings.

LeDoux is hesitant in using interpretive terms such as “emotions” and “feelings” and advocates “survival circuits” both as a term (LeDoux, 2017) and as a focus in animal research (LeDoux, 2014). Animals may have emotions the way Anderson and Adolphs describe them, but due to unique features of the human brain (e.g., Koechlin, 2011; Preuss, 2011; Semendeferi et al., 2011), other species lack the same consciousness and linguistic distinctions as humans, making it questionable whether they can have feelings like us. LeDoux does not say that animals cannot have feelings. According to him, emotions are not the cause of feelings. Rather, survival circuit activity occurs in parallel as a correlate to, rather than as the cause of conscious feelings (LeDoux, 2021). This makes it impossible to know about feelings without asking about them directly.

In biology, finally, views on emotions go back to Darwin’s (1872) *The Expression of the Emotions in Man and Animals*, which stressed continuity between humans and other species. As exemplified by de Waal (2011, 2019), emotions are considered species-typical and placed in an evolutionary light as they prepare the organism for adaptive behavior. Everything about them, including the possibility of feelings, is assumed to be similar between related species even if the focus of the behavioral biologist is always more on emotional behavioral expression than on internal states. Thus, de Waal (2011, p. 201) takes a position like Anderson and Adolphs (2014), saying about animals that similar feelings may be assumed but that the actual experiences of animals remain mostly inaccessible for the time being.

In sum, most researchers agree that animals show responses to certain stimuli that are adaptive and based on internal states that may or may not be referred to as emotions. In contrast, there is considerable discussion about whether animals have conscious awareness of these emotions – feelings – and how important feelings are in relation to said emotions.

## Our View on Emotions and Feelings in Humans and Other Animals

Our position is that the exclusion of subjective experiences of emotions in other animals is highly unreasonable (for similar positions, see, e.g., Burghardt, 2019; Bekoff & Pierce, 2017; Paul et al., 2020). Importantly, subjective experiences probably differ between species and also between individuals within a species. Every species has evolved under specific environmental selection pressures and has a body and a brain that is unique in its form and output. Some species, however, such as chimpanzees and humans, are relatively similar, since they share a long evolutionary history. The way emotions manifest themselves in humans and apes is also very similar, including homologous facial expressions that activate a facial musculature that is nearly identical between humans and chimpanzees (Burrows et al., 2006; Parr et al., 2007). Logically, given the similarity in body and brain, the same may hold for how emotions are being experienced as feelings by the members of closely related species.

As for feelings, we know introspectively that we experience them ourselves. However, this is the only direct evidence that we have. Feelings are not visible from the outside, which is why they are often being denied in nonverbal organisms. Remember that there was a time when people believed the same about the feelings of human neonates. As a consequence, neonates were, for example, operated upon (e.g., circumcision) without anesthesia. Nowadays, most people would agree that this was an incorrect assumption even though the body and brain of a young infant are very different from those of adults, sometimes even more so than those of closely related but different species. If we could ask a naïve extraterrestrial which two out of three individuals, human adult, human infant, and adult chimpanzee are most similar, we are not sure that the alien would point at the two humans in this trio. There exist numerous areas in which the differences between humans (e.g., young vs. old or brain-damaged vs. neurotypical) are larger than those between closely related species, but we don’t deny any humans feelings.

We not only believe that various animal species experience feelings but also that they may experience unique feelings due to their unique evolutionary background. Feelings are most likely similar if the evolutionary pathways overlap. Similar to the unique evolutionary pathways of different species, individuals within a species all have their own developmental pathways that shape body and brain in form and output. Consequently, we expect intraspecific variation in how feelings manifest themselves. Hence our title that “My fear is not, and never will be, your fear.” We will never know what it is like to be a bat (Nagel, 1974), but neither will we know exactly what it is like to be our neighbor.

Feelings are hard to demonstrate in other species since they cannot verbally report their inner states. However, it would be a mistake to think that humans always know what they feel. Many people visit a therapist to figure that out. Further, people likely respond in ways they deem socially desirable (e.g., “I’m fine”), which makes it hard to measure actually felt feelings in humans.

## Our View on Measuring Emotions and Feelings

First and foremost, when aiming to investigate emotions or feelings, the terms should be clearly defined an operationalized. In the relevant literature, different terms are being used to refer to emotion. Some researchers choose to use certain terms exclusively for humans, while other words are mostly used for other animals. Andrews (2020) stresses that if we invent new words for other species while keeping old words for human beings, we are throwing up unnecessary semantic barriers to comparing humans with other species. Interpreting behavior as associated with a particular feeling in humans and interpreting the same behavior as something else in animals thwarts comparative research, and consequently the progress of fundamental research into the proximate and ultimate causations of emotions.

Increasingly, researchers approach emotions as multifaceted states that include physiological, behavioral, and cognitive components that are measurable (Mendl et al., 2010; Paul et al., 2005; Massen et al., 2019). We believe that insight in emotion states can be gathered with various methods.

Numerous examples could be given, but just to make our case, we will cover a few here that combined relatively noninvasive methods in various species to compare behavioral and physiological measures of emotions. In a study using thermography, Nakayama et al. (2005) demonstrated a decrease in nasal skin temperature in rhesus macaques in response to a human dressed in a lab coat and holding a catching net. Along with this temperature drop, the monkeys frequently showed a silent bared-teeth face, staring open-mouth face, and lip-smacking, all expressions of negative emotions. Another example is the finding that in a touchscreen task, bonobos had an attentional bias towards emotional expressions of conspecifics. In addition, more “nose-wipes” were observed during trials where an emotional image had to be approached rather than avoided, indicative of emotional arousal (Kret et al., 2016). In an experiment with dogs, it was shown that when separated from their owner, dogs were more alert. They stood up, walked, or ran around and especially towards the door, while barking and whining. On the physiological level, a detailed analysis of their heart rate suggested a negative emotion (Katayama et al., 2016). Another study by combining behavioral and cardiac measurements suggests that sheep have negative emotions following negative events, and positive emotions following positive situations (Reefmann et al., 2009). Cardiac activity and salivary cortisol concentration were combined in a study with horses (Janczarek et al., 2019). The horses showed negative emotions in response to the presence of an audience in the arena. By measuring psychophysiological reactions, hormone levels, cognitive bias tasks or behavioral observations, emotions can be inferred.

Emotions are contagious: i.e., they easily spread throughout a social group. Various studies have shown basic forms of empathy in social species, from rodents to primates, such as mimicry of expressions of emotion, matching another’s emotional state, and responding to the distress of others with reassurance behavior or helping actions (reviewed by Preston & de Waal, 2002; de Waal & Preston, 2017). Some animals, such as ravens, not only match conspecifics’ emotions on a behavioral level (e.g., Osvath & Sima, 2014) but also match their judgement bias, which is interpreted as an emotional state, after having witnessed a conspecific react with apparent frustration to a negative manipulation (Adriaense et al., 2019). Chimpanzees show jealous reactions when their own valuable social bonds are under threat (Webb et al., 2020); long-tailed macaques relax (i.e., show a decrease in circulating cortisol) while cooperating with a friend (Stocker et al., 2020), and several species consider “the glass half full rather than half empty” in judgement- or cognitive bias tasks (Paul et al., 2020). In line with Panksepp’s argument, recent research incorporating the behavioral, physiological, and cognitive components of emotions, is thus suggesting that not only do animals show emotional behavioral responses, they also seem to experience them as well as those of conspecifics (Kret et al., 2020; Nieuwburg et al., 2021).

Emotions are embedded in a complex network of brain structures including both cortical and subcortical areas activated in close interplay with the body (Prochazkova & Kret, 2017). Recent neuroimaging research taps into emotions and their cognitive interpretation and shows that it’s too simple to state that “feelings are cortical.” Using ecologically valid paradigms involving risky decisions or social dilemmas to induce strong emotions, these studies have shown the pivotal role of ancient brain structures in human feelings of social exclusion, depression, and even suicidal tendencies (e.g., Cáceda et al., 2020). Other studies have demonstrated a close connection between the body and the brain. For example, a study put participants under high levels of stress while measuring bodily responses (heart rate, skin conductance, cortisol), self-reported stress levels, and brain activity via fMRI. Clear relationships were observed between the neural responses on the one hand and bodily responses and self-reported stress on the other (Orem et al., 2019).

Emotions even recruit the most ancient neural structures. The spinal cord rapidly activates in response to emotional stimuli (Smith & Kornelsen, 2011), and classical work by Hohmann (1966) has shown that a spinal cord lesion drastically impacts the feelings reported by patients. We are not saying here that all animals with a spinal cord have feelings. But the involvement of such structures, which are highly conserved among vertebrates (Leung & Shimeld, 2019), casts doubt on the emphasis on consciousness, language, cultural construction, and human uniqueness. Even the evolutionarily more “recent” neural architecture of humans is mostly shared with mammals and birds (e.g., Stacho et al., 2020). The human brain is hardly categorically distinct from other brains. That said, we don’t deny that the human brain may have features that other species lack (e.g., Koechlin, 2011; Preuss, 2011; Semendeferi et al., 2011) and that these unique structures may alter emotional experiences (LeDoux, 2017). At the same time, this is also true for other species since all species have unique brains. The difference is that there is a lot more unknown about the role of other species’ brain structures in emotions. Demonstrating parallel neural mechanisms involved in the emotions of humans and other animals, Panksepp (2011) saw no reason to postulate different emotional experiences, and we tend to agree with him on this point.

But even if we do put an emphasis on consciousness: Recent studies on mirror self-recognition (as a proxy for self-awareness), theory of mind, metacognition, and planning for the future (reviewed by de Waal, 2016) do suggest self-reflective capacities in animals, even if some other scientists remain skeptical (e.g., Heyes, 2017; Povinelli, 2020). For example, rhesus monkeys have “memory awareness” in that they know what they know or don’t know (Smith et al., 2013; Templer & Hampton, 2012); capuchin monkeys, California scrub jays, and Eurasian jays seem to not only grasp what others know, but also what others desire (Hattori, 2012; Ostojić et al., 2017), and chimpanzees take (false) beliefs of conspecifics into account (Krupenye et al., 2016). Given how ill-defined consciousness is and how widespread advanced cognitive traits seem to be in other species, we deem it premature to assume that said species have no consciousness of their emotions. From an evolutionary perspective, it is more logical to assume humanlike consciousness in species related to us rather than deny it, which means that we best adopt the former as a working hypothesis (de Waal, 2019).

### Cognitive and Evolutionary Parsimony and Ethical Considerations

Imagine an animal that backs away from a harmful stimulus. We see a chimpanzee who, while staring at a snake and uttering soft alarm calls, carefully and slowly moves out of the way. The starting point of some scientists, for example, LeDoux, is that such behavior should be assumed to be unconsciously controlled and devoid of feelings unless proven otherwise. The rule of *cognitive parsimony* is applied here, which postulates the simplest possible cognitive process when it comes to interpretating behavior. Cognitive parsimony is important to consider when interpreting behaviors of many animal species. If in the example above we would have described the behavior of a fruit fly instead of a chimpanzee, we agree that this approach would be most correct. However, the example is about humans’ most closely living relative, a species that is well-studied and one that we have a lot of information on.

It is important to realize that the rule of cognitive parsimony is rarely applied to human behavior. When researchers suggest that human emotions rely on higher-order conscious cognitive processes, since humans verbally report their emotions, they risk postulating processes that may be unnecessary, hence violating Occam’s razor. Setting aside the discussion about the validity of self-report, feelings are best considered the consequence rather than the cause of emotional states (Anderson & Adolphs, 2014). Consequently, we think that feelings cannot inform us about the cognitive processes and complexity of the emotional state itself. In our view, this chimpanzee in the above example is feeling scared and is acting deliberately cautiously to minimize potential harm. This does not mean that that interpretation is all that should be reported. To get the picture complete, it should be accompanied with an objective description of the behavior so that this data remains accessible and open for future interpretation.

By looking to preserve *cognitive* parsimony at all costs, comparative psychologists may be disregarding *evolutionary* parsimony, which dictates that we should offer explanations that posit the fewest possible changes in the phylogenetic tree. Although cognitive parsimony can be important, we here would like to emphasize the biological stance of *evolutionary parsimony*, stating that when related species show similar behavior under similar circumstances, these are likely driven by similar psychological processes (de Waal, 1999). Until the contrary can be demonstrated, we must assume that similar behavior in these species is paired with similar emotions and in some cases similar feelings. This position is, of course, not entirely new. One of the first to advocate cross-specific uniformity in behavioral explanations was philosopher David Hume (1739, p. 226), who formulated the following touchstone well before we had a theory of evolution:

Tis from the resemblance of the external actions of animals to those we ourselves perform, that we judge their internal likewise to resemble ours; and the same principle of reasoning, carry'd one step further, will make us conclude that since our internal actions resemble each other, the causes, from which they are deriv'd, must also be resembling. When any hypothesis, therefore, is advanc'd to explain a mental operation, which is common to men and beasts, we must apply the same hypothesis to both.

Hume’s stance raises the question how far we can stretch the concept of similar states and how related two species should be for them to experience similar feelings. Whereas, given the ancient structures and mechanisms involved, we do expect some sort of emotional states in all animals, we are agnostic about feelings in distantly related species, such as invertebrates. Conversely, we argue that it is unreasonable to exclude the possibility of feelings in *all* animals and specifically in those that are closely related to us, hence similar in body and brain. We, furthermore, embrace the idea that feelings may have evolved convergently in multiple lineages and that the comparative study of these taxa will help us shed light on the selection pressures that may have shaped the evolution of both emotions and feelings (Fitch et al., 2010; Massen, 2020).

Finally, we would like to include a warning. Those who do not set emotions apart from feelings, and doubt the latter’s existence in animals, have a special obligation to produce convincing evidence when they deny the existence of animal feelings. This Cartesian position carries ethical implications. Humans experience a different sense of obligation towards entities with or without feelings, which is why the question of animal *sentience* is central to every current debate about the humane treatment of animals. This means that we need to proceed with the utmost care in this domain so as to avoid giving fodder to those who consider animals unworthy of moral consideration. We (including the authors of this opinion piece) have an obligation to be clear about what is a mere assumption and what is fact when it comes to animal feelings. At the same time, scientific evidence of animal emotions is needed to create a better understanding of the depth of their emotional lives. To that extent, the section below lists some important steps to be taken.

### Future Steps

#### Where to Go from Here?

First, we should shift the focus from things we cannot measure to things that we *can* measure (Adolphs & Andler, 2018). For example, technological advances allow us to measure animal’s emotional facial expressions (e.g., the chimpanzee’s and other species’ FACS of Waller et al., 2020). We can also noninvasively measure bodily expressions and physiological arousal by using thermography, pupillometry, heartrate measurements, hormone levels, and measurements of neural activation (reviewed by Nieuwburg et al., 2021). Similarly, we study emotionally biased decision-making and the perception of emotions in experimental paradigms with techniques such as touchscreens and eyetrackers (e.g., Parr & Heintz, 2009; Kret et al., 2016). We acknowledge that the associated feelings still remain inaccessible but note that this also largely holds for humans. For this reason, our recommendation to focus on emotions’ measurable aspects extends to modern psychological research on humans, which thus far has concerned itself more with feelings than emotions. Doing so will facilitate comparisons between humans and other species and move us away from the unreliability of introspection (Baumeister et al., 2007). This is not to deny the importance of feelings, but there is debate (see above) about how essential they are to the way emotions work.

Second, the aim of human and comparative psychology is to understand psychological states or traits through experimentation, observation, and interview. We seek to understand behavior and assign meaning to what we see, often using hypotheses based in physiology, neuroscience, and/ or evolutionary theory. Whether postulated intervening variables are knowable or unknowable is not always the issue. We don’t ask astronomers not to invoke gravity, which is invisible, to explain planetary movements, or biologists not to invoke shared evolution, which is also invisible, to explain why chimpanzee hands are so strikingly similar to those of humans. Science is full of postulated intervening variables to make sense of observed phenomena. In the same way, the invisibility of animal feelings is not a good argument against them.

A third step forward, in our view, is to try to take the perspective of animals more when asking questions and designing studies. If we take a typically human phenomenon and ask the question whether, say, chimpanzees show it too, it is more likely that this behavior characterizes us better than them. The animal behavior literature is full of examples where we have misjudged animals based on human testing biases (de Waal, 2016). These biases often dictate the search for humanlike traits in animals, especially in those that are closely related to us, and in doing so overlooks the uniqueness of other species. We have trouble seeing a chimpanzee the way a conspecific does. Rather than focusing on humanlike emotions, we should consider the species-specific emotions of other animals as they have evolved in line with that species’ specific needs. We need a bottom-up approach that does not necessarily focus on predefined human emotions.

To conclude, in our view, if a species shows behavioral, neurophysiological, hormonal, or cognitive responses to valenced stimuli, we can speak of emotions until proven otherwise. In some instances, we might even speak of feelings. We advocate a multi-method rather than a single method approach and believe that the variety of species that we can study, with their unique brains and bodies, can give us new insights into emotions and feelings.

## Footnotes

1 Panksepp used the word “emotional feelings.” In his view, core emotional feelings may reflect a variety of extended emotional action systems—including seeking, fear, rage, lust, care, panic, and play. In the current article, we try to keep the number of different terms used in the literature to a minimum.
